# Editorial: Vector-borne diseases and consequences on human health: a multidisciplinary approach

**DOI:** 10.3389/fpubh.2023.1326243

**Published:** 2023-11-13

**Authors:** Ahmed Tabbabi, Thiago Vasconcelos dos Santos, Khadija Bekhti, Najoua Haouas, Pang Junxiong, Miguel Angel Garcia-Bereguiain

**Affiliations:** ^1^Division of Medical Zoology, Department of Infection and Immunity, Jichi Medical University, Shimotsuke, Tochigi, Japan; ^2^Parasitology Department, Evandro Chagas Institute (Secretary of Health and Environment Surveillance, Ministry of Health), Ananindeua, Pará, Brazil; ^3^Microbial Biotechnology and Bioactive Molecules Laboratory, FST, University of Sidi Mohammed Ben Abdeallah, Fes, Morocco; ^4^Laboratory of Medical and Molecular Parasitology-Mycology LP3M (Code LR12ES08), Department of Clinical Biology B, Faculty of Pharmacy, University of Monastir, Monastir, Tunisia; ^5^Centre for Outbreak Preparedness, SingHealth Duke-NUS Global Health Institute, Duke-NUS Medical School, National University of Singapore, Singapore, Singapore; ^6^One Health Research Group, Universidad de Las Américas, Quito, Ecuador

**Keywords:** vector-borne diseases, pathogens, hosts, vectors, climate changes, susceptible human populations

Emerging and re-emerging vector-borne diseases are among the major public health concerns across the world. They are not directly transmissible among humans in most cases, but are mainly spread under suitable conditions as a result of the interaction of vectors, hosts, climate/environment, pathogens, and vulnerable human populations. Their impact has a significant toll on economies and restricts both rural and urban development. Burden is the highest in tropical and subtropical areas, and they disproportionately affect the poorest population.

This editorial summarizes collections on the Research Topic “*Vector-borne diseases and consequences on human health: a multidisciplinary approach*” which invites the latest research findings on these (re-) emerging diseases. Eleven articles from researchers across the globe (China, India, Japan, Iran, Malaysia, Argentina, Ecuador, Canada, and Sweden) were published.

The effect of global warming on public health and, more particularly, on the seasonal and geographical expansion of pathogens is still increasing due to the propagation of vectors. A systematic literature review and meta-analysis was conducted into the impacts of temperature on dengue vectors and authors revealed various data on the life cycle of *Aedes* mosquitoes at diverse temperatures which may be used for effective dengue management in Malaysia (Halim et al.). In China, the boosting regression tree model was used to explore the intrinsic relationship of *Scrub typhus* (a bacterial disease transmitted by mites) with meteorological, environmental and social factors. Many counties in the Sichuan Province were evaluated to be receptive to *Scrub typhus*. Their findings may achieve the priorities of disease control and prevention to manage precise and effective allocation of resources (Zhang et al.). In the same context, a literature review focused on the use of risk maps to model distributions of *Borrelia burgdorferi* and its vector, the blacklegged tick *Ixodes scapularis*, in North America to compare variables employed to predict these spatial models (Fellin et al.). Environmental variables were classified to be the most widely used in risk spatial models, specifically temperature. Limited anthropic variables were considered, mainly in studies that predict future risk, although the purpose of these models directly or indirectly concentrate on health intervention strategies. Authors indicated that it is really hard to estimate how reliable these risk maps truly are without including human-related factors and taking into consideration these variables within risk map models.

In a grand challenges paper on vector borne diseases, many authors focused on the incidence and the spread of these (re-) emerging diseases. A countrywide geodemographic epidemiological analysis from 2011 to 2021 of severe Chagas disease (transmitted by triatomine species) in Ecuador was investigated by Vásconez-González et al.. A total of 118 patients have been hospitalized in Ecuador during this period due to Chagas disease. The overall in-hospital mortality percentage was 69.4%. The incidence rate of men was higher than women, despite women having a remarkably higher mortality rate than men which seemed to be more infected due to differences in work and sociocultural habits. Authors mentioned also the role of environmental change in the proliferation of disease-carrying vectors in previously unaffected areas. Several electronic databases were used to select articles treating malaria in pregnancy in India and its results on both mother and child (Foko and Singh). Their results showed that malaria in pregnancy was mostly due to *Plasmodium falciparum* and *P. vivax*, and rarely to *P. ovale* and *P. malariae*. The overall prevalence of malaria in pregnancy was ranging from 0.1 to 57.7% for peripheral malaria and from 0 to 29.3% for placental malaria. According to Wilkman et al., five different mosquito-borne viruses involved in human disease are well-known in Fennoscandia including Sindbis virus, Inkoo virus, Tahyna virus, Chatanga virus, and Batai virus. However, the incidence of mosquito-borne virus infections is still unknown, mainly due to underdiagnosing and lack of control efforts. Early detection of invasive viruses, would be remarkably supported by collaboration between clinicians and other key players applying a One Health approach as recommended by the authors. In the same context of incidence in vector borne diseases, Calvopiña et al. identified the first case caused by the zoonotic *Babesia bigemina* (responsible for a tick-borne disease known as babesiosis) in the Amazon region of Ecuador ensuring the existence of active transmission. The authors highlighted the need for alerting public health decision-making authorities on the emergence of this zoonosis and the need for research to determine strategies to reduce tick exposure.

Substantial progress has been made against many vector-borne diseases during the past decade. In this context, analyses of the epidemiological data during 1989–2020 indicated that Iran has made stable advance and remarkable progress over the past decade, yet persistent challenges exist to decrease the cutaneous leishmaniasis burden in the country (Sharifi et al.). Well-trained staff and experienced clinical practitioners should reinforce country-level capacity-building to support effective control strategies across the healthcare system as indicated by the authors.

Resistance to insecticides persists a major barrier not only for efficient control of malaria but also other vector-borne diseases. Fay et al. reported the first report of knockdown resistance (kdr) mutations in *Aedes aegypti* in the northeast region of Argentina. Authors demonstrate the relevance of kdr information for focused vector control interventions and public health initiatives to minimize both *Aedes aegypti* populations as well as insecticide use/misuse to reduce arboviral disease risks. In Xiamen city of China, a propagation dynamics model was used by Guo et al. to evaluate and estimate the risk of dengue fever. Authors emphasized that imported cases, community population, mosquito density and insecticide resistance have an important role in the transmission of local dengue fever. However, mosquito resistance to insecticide has been identified as the most serious variable for assessing dengue fever communication risks and applying management control systems.

The Research Topic of articles highlights some crucial advances in controlling mosquito-borne diseases, which will contribute to the development of new appropriate methods and tools in vector control. An example of such advances is the report of allose, a rare sugar, to inhibit the development of *Plasmodium* parasites in laboratory-reared *Anopheles* mosquitoes. As a first step to address the inhibition mechanism, Mizushima et al. revealed the non-involvement of the midgut microbiota in the recorded inhibition of *Plasmodium* parasites in the mosquito. This sugar may be a useful material for vector control of malaria as a “transmission-blocking sugar” in the future.

This Research Topic of articles covers ticks, mosquitoes, sand flies, mites, and triatomines bugs-borne pathogens through different continents which indicates their wide distribution and reveals the diversity of hosts and pathogens. An integrated One Health approach would promote a better understanding of the intrinsic complexity of disease transmission emphasizing interactions between human, animal, environmental health, and the importance of transdisciplinary efforts.

In conclusion, we hope that the papers collected in this Research Topic will expand the knowledge base and skills related to vector-borne diseases, particularly in the context of political, socioeconomic, environmental, and climate change factors, enabling the development of practicable solutions both at local and global scales.

## Author contributions

AT: Writing – original draft, Writing – review & editing. TVS: Writing – review & editing. KB: Writing – review & editing. NH: Writing – review & editing. PJ: Writing – review & editing. MG-B: Writing – review & editing.

